# Hepcidin-induced reduction in iron content and PGC-1β expression negatively regulates osteoclast differentiation to play a protective role in postmenopausal osteoporosis

**DOI:** 10.18632/aging.202817

**Published:** 2021-04-04

**Authors:** Hui Zhang, Aifei Wang, Guangsi Shen, Xiao Wang, Gongwen Liu, Fan Yang, Bin Chen, Mingyong Wang, Youjia Xu

**Affiliations:** 1 Department of Orthopaedics, The Second Affiliated Hospital of Soochow University, Suzhou 215004, China; 2 Osteoporosis Clinical Center, The Second Affiliated Hospital of Soochow University, Suzhou 215004, China; 3 Institute of Osteoporosis Diagnosis and Treatments of Soochow University, Suzhou 215004, China; 4 Department of Orthopaedics, Suzhou TCM Hospital Affiliated to Nanjing University of Chinese Medicine, Suzhou 215004, China

**Keywords:** ROS, iron, postmenopausal osteoporosis, hepcidin, PGC-1β

## Abstract

As a necessary trace element, iron is involved in many physiological processes. Clinical and basic studies have found that disturbances in iron metabolism, especially iron overload, might lead to bone loss and even be involved in postmenopausal osteoporosis. Hepcidin is a key regulator of iron homeostasis. However, the exact role of hepcidin in bone metabolism and the underlying mechanism remain unknown. In this study, we found that in postmenopausal osteoporosis cohort, the concentration of hepcidin in the serum was significantly reduced and positively correlated with bone mineral density. Ovariectomized (OVX) mice were then used to construct an osteoporosis model. Hepcidin overexpression in these mice significantly improved bone mass and rescued the phenotype of bone loss. Additionally, overexpression of hepcidin in OVX mice greatly reduced the number and differentiation of osteoclasts *in vivo* and *in vitro*. This study found that overexpression of hepcidin significantly inhibited ROS production, mitochondrial biogenesis, and PGC-1β expression. These data showed that hepcidin protected osteoporosis by reducing iron levels in bone tissue, and in conjunction with PGC-1β, reduced ROS production and the number of mitochondria, thus inhibiting osteoclast differentiation and bone absorption. Hepcidin could provide new targets for the clinical treatment of postmenopausal osteoporosis.

## INTRODUCTION

Osteoporosis is a disease that causes a decrease in bone mineral density (BMD) due to various reasons. In menopausal women, the rapid loss of bone mass after 5-10 years of menopause is called postmenopausal osteoporosis [1]. Postmenopausal osteoporosis is characterized by the activation of osteoclasts, mainly due to estrogen deficiency [2, 3]. Estrogen is a strong inhibitor of differentiation and function in osteoclasts. Estrogen deficiency causes the expression of the estrogen-induced receptor activator for nuclear factor-κβ ligand (RANKL) antagonist osteoprotegerin (OPG) to decrease, resulting in proliferation and activation of osteoclasts through the RANKL/OPG pathway [4, 5]. In addition, estrogen receptors (ERs) also bind to nuclear factor-κβ (NF-κβ), inhibiting NF-κβ signaling pathway activity and osteoclast differentiation [6, 7]. During estrogen deficiency, the bone resorption biochemical index is increased by 90%, while bone formation is increased by only 45% [8, 9]. Since bone resorption is far greater than bone formation, osteoporosis sets in, increasing the risk of fragile bones and fractures.

Iron is an essential trace element in the human body. With the reduction of iron elimination in postmenopausal women, the level of ferritin, one of the storage forms of iron, increases in the body [10]. Studies have found that iron, as an independent risk factor for osteoporosis, can induce bone loss by accumulating in the body, which may be one of the reasons for accelerated bone loss after menopause [11, 12]. In mature osteoblasts, iron inhibits osteoblast bone remodeling by inhibiting bone morphogenetic protein 2 (BMP-2) and Wnt signaling pathway [13, 14]. A mouse model of iron accumulation was generated in 2010 by injected ferric ammonium citrate (FAC) which is commonly used in the body to supplement iron [15]. We, in a similar manner, constructed an iron accumulation mouse model [16], and found that in the case of estrogen deficiency, iron could significantly accelerate bone loss. Further research found that increased reactive oxygen species (ROS) produced by iron accumulation could promote osteoclast differentiation through the NF-κB signaling pathway [17]. Peroxisome proliferator-activated receptor gamma coactivator-1 (PGC-1) is a co-activator of peroxisome proliferator activated receptor gamma γ (PPARγ). This complex is widely involved in multiple metabolic pathways, such as promoting nuclear respiratory factor (NRF) and other mitochondrial related genes to promote mitochondrial biogenesis. Studies have found that iron produces ROS to activate peroxisome proliferator-activated receptor gamma coactivator-1β (PGC-1β) expression and increase mitochondrial biogenesis, and thereby promote osteoclast differentiation [18, 19].

Hepcidin is an essential regulator of system iron homeostasis. It was first discovered by Sayre et al. in 2000 [20]. Hepcidin is an iron reducing hormone mainly synthesized and secreted by the liver and excreted in the urine (macrophages can also synthesize and secrete it in small amounts) [21]. At present, ferroportin 1 (FPN1) is the only known iron export membrane protein on the surface of target cells. Hepcidin interacts with FPN1 and then internalize and degrade the latter to reduce the absorption of iron from the intestines and increase iron storage in organs, such as liver and spleen (cells), to reduce the circulating iron content [22–24]. In hepcidin-mutated mice, ferritin level was elevated and bone loss was increased [25, 26]. Using the MO knockdown zebrafish hepcidin model, we found that bone mineralization and bone formation were significantly reduced, and overexpression of hepcidin reduced the bone loss caused by iron accumulation [27]. However, protection of postmenopausal bone loss by hepcidin overexpression has not been reported. In this study, tamoxifen-induced conditioned hepcidin overexpression in transgenic mice was used in a postmenopausal osteoporosis mouse model by performing ovariectomy. The aim of this study was to induce hepcidin overexpression to observe the protective effects of hepcidin in postmenopausal osteoporosis, and further provide new targets for the clinical treatment of postmenopausal osteoporosis.

## RESULTS

### Serum hepcidin levels were decreased in postmenopausal osteoporosis patients

**Figure 1:**
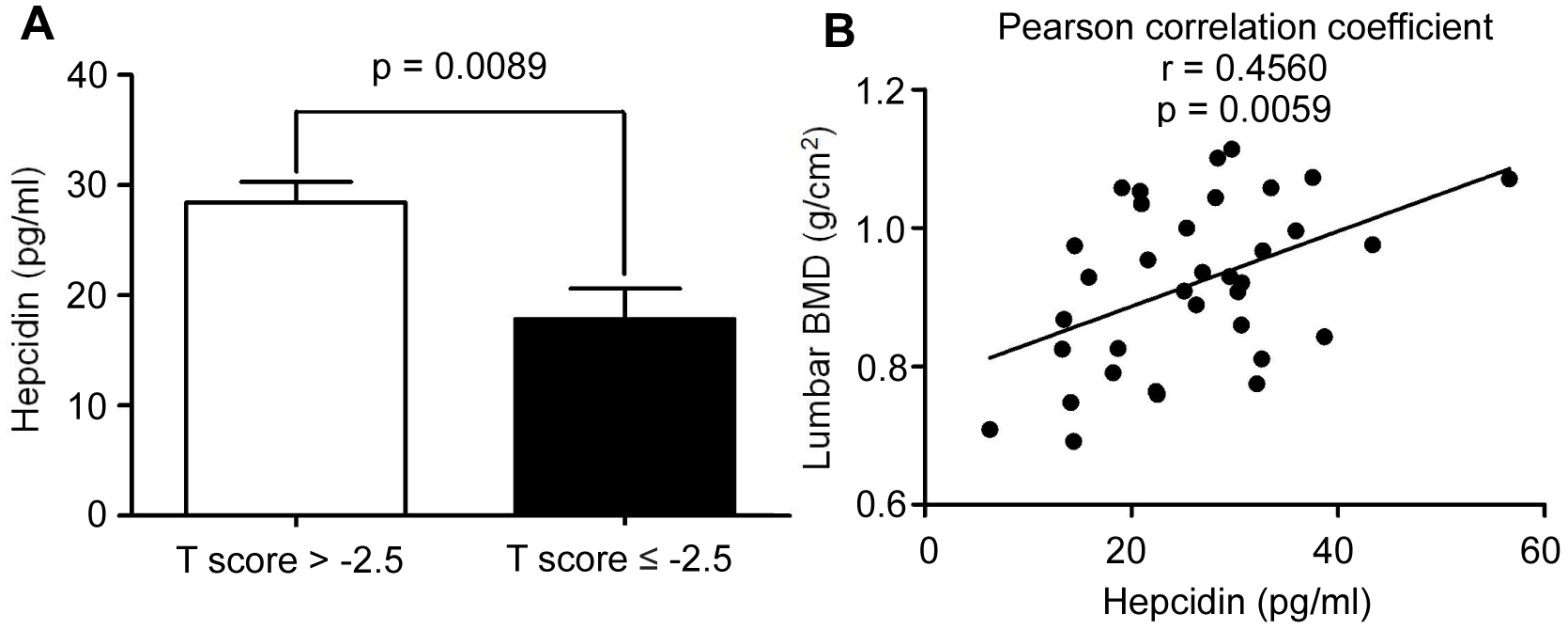
**The hepcidin content is decreased in the postmenopausal women with osteoporosis.** (**A**) Hepcidin concentration in the serum of peripheral blood in the postmenopausal women with osteoporosis (T score ≤ 2.5); (**B**) Lumbar BMD and serum hepcidin concentration showed a positive linear correlationship in the postmenopausal women with osteoporosis; r = 0.4560; statistical significance was considered at P = 0.0059, (n = 35; age = 62.11 ± 5.9).

Previous studies found that iron accumulation is a negative regulator of bone metabolism. Hepcidin is an important regulator of iron metabolism. However, how changes in the concentration of hepcidin affect postmenopausal osteoporosis, is unknown. In order to explore this problem, the serum of 35 postmenopausal women undergoing physical examination related to iron metabolism and bone metabolism were collected. The ages of candidates were 62.31 ± 5.79 years, with average serum hepcidin and ferritin concentrations being 26.01 ± 10.35 (pg/mL) and 205.13 ± 98.39 (ng/mL), respectively. The lumbar spine and femoral neck bone mineral density (BMD) levels were reported as 0.92 ± 0.12 (g/cm^2^), and 0.79 ± 0.11 (g/cm^2^), respectively. All other data have been detailed in Supplementary Table 1. All patients were divided into two groups based on BMD: the osteoporosis group (T-score ≤ -2.5) and the non-osteoporosis group (T-score > -2.5). Comparing the data of the two groups, it was found that serum hepcidin in the osteoporosis group (T-score ≤ -2.5) was significantly lower than that in the non-osteoporosis group (T-score > -2.5) (p < 0.05) (Figure 1A). Linear regression analysis of serum hepcidin and lumbar spine BMD revealed that there was a significant positive correlation between serum hepcidin and lumbar spine BMD (Pearson r = 0.4560, p = 0.0059) (Figure 1B). These results indicated that a positive correlation between serum hepcidin and BMD in postmenopausal women.

### Hepcidin overexpression rescued bone loss induced by ovariectomy (OVX) in mice

**Figure 2:**
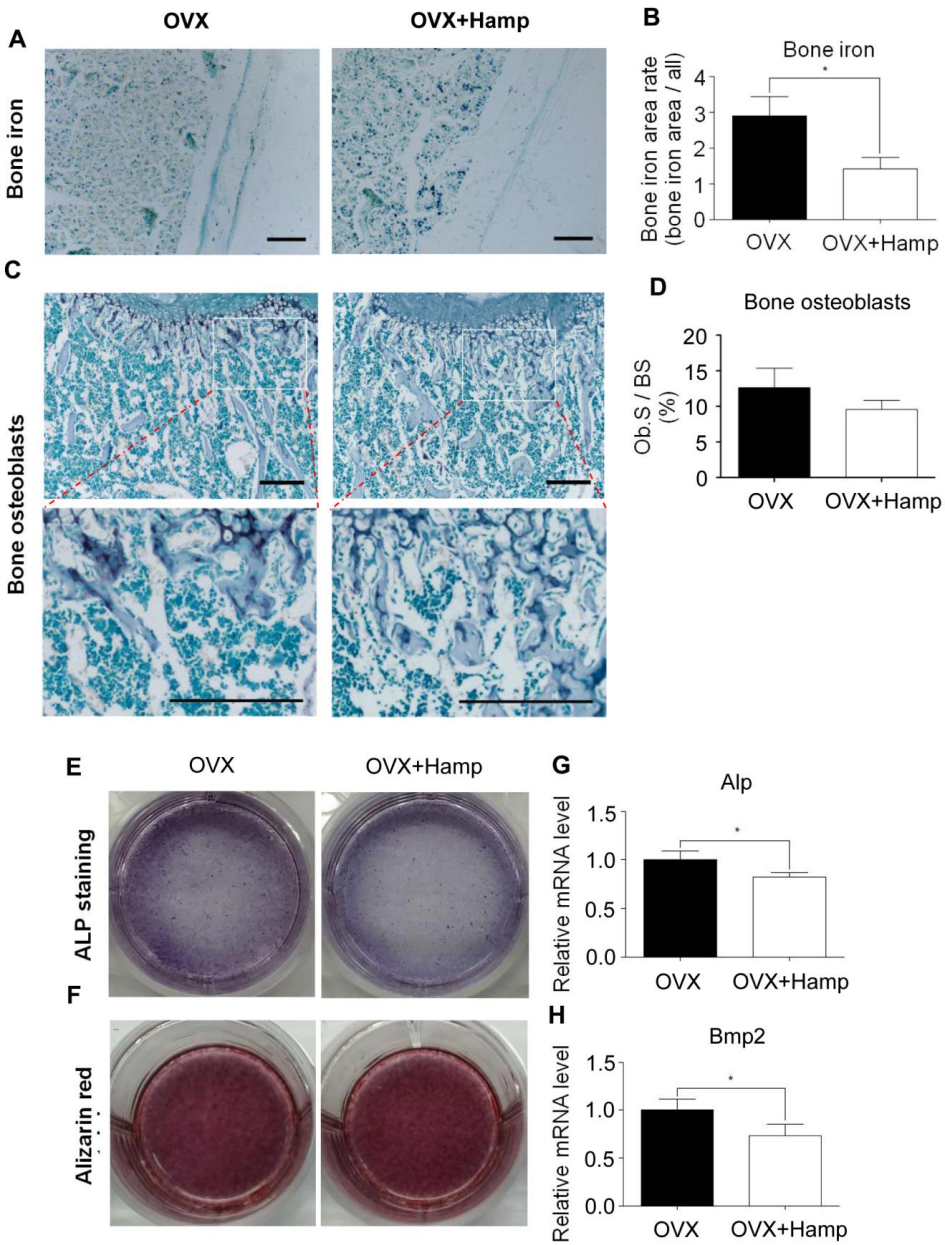
**Hepcidin overexpression has little effect on osteoblasts in the OVX mouse.** (**A**, **B**) Hepcidin overexpression decrease the irons contents in the mouse bone. (**C**, **D**) ALP stain shows that overexpression Hepcidin does not significantly inhibit the number of osteoblasts in femoral bone in the OVX mouse; (**E**, **F**) Osteoblasts, which cultured by mouse serum for 14 or 21 days, stained with ALP or alizarin red to assess its differentiation and mineralization; (**G**, **H**) Quantitative polymerase chain reaction (q-PCR) analysis of the expression of bone formation markers including Alp and Bmp2. Scale bar, 200 μm. The asterisks (*) indicate significant differences at P < 0.05.

To further study the effects of hepcidin on postmenopausal bone metabolism, hepcidin overexpression mice were used to study the effect of hepcidin on postmenopausal bone loss. Mice conditionally overexpressing hepcidin were bilaterally ovariectomized to construct a postmenopausal osteoporosis model. Then, tamoxifen was injected intraperitoneally into the model mice to induce hepcidin overexpression. To rule out the effect of tamoxifen on bone mass in mice, two groups of experimental mice were injected intraperitoneally with an equal amount of tamoxifen. It was found that the levels of estrogen in the two groups of mice decreased rapidly by OVX (Supplementary Figure 1A). The concentration of hepcidin was higher, while ferritin was lower significantly in the serum of overexpressing mice (Supplementary Figure 1B, 1C). In order to observe whether overexpression of hepcidin affected iron metabolism, liver and undecalcified tibia sections were stained with Perls staining. The results demonstrated that the iron content in the liver increased significantly (Supplementary Figure 1D, 1E), but the iron content in bone surface decreased significantly in the tamoxifen induced group (Figure 2A, 2B). These data indicated that hepcidin regulated iron distribution and storage in the liver, an iron storage organ. Therefore, the available iron content in the circulation and bones was decreased.

**Figure 3:**
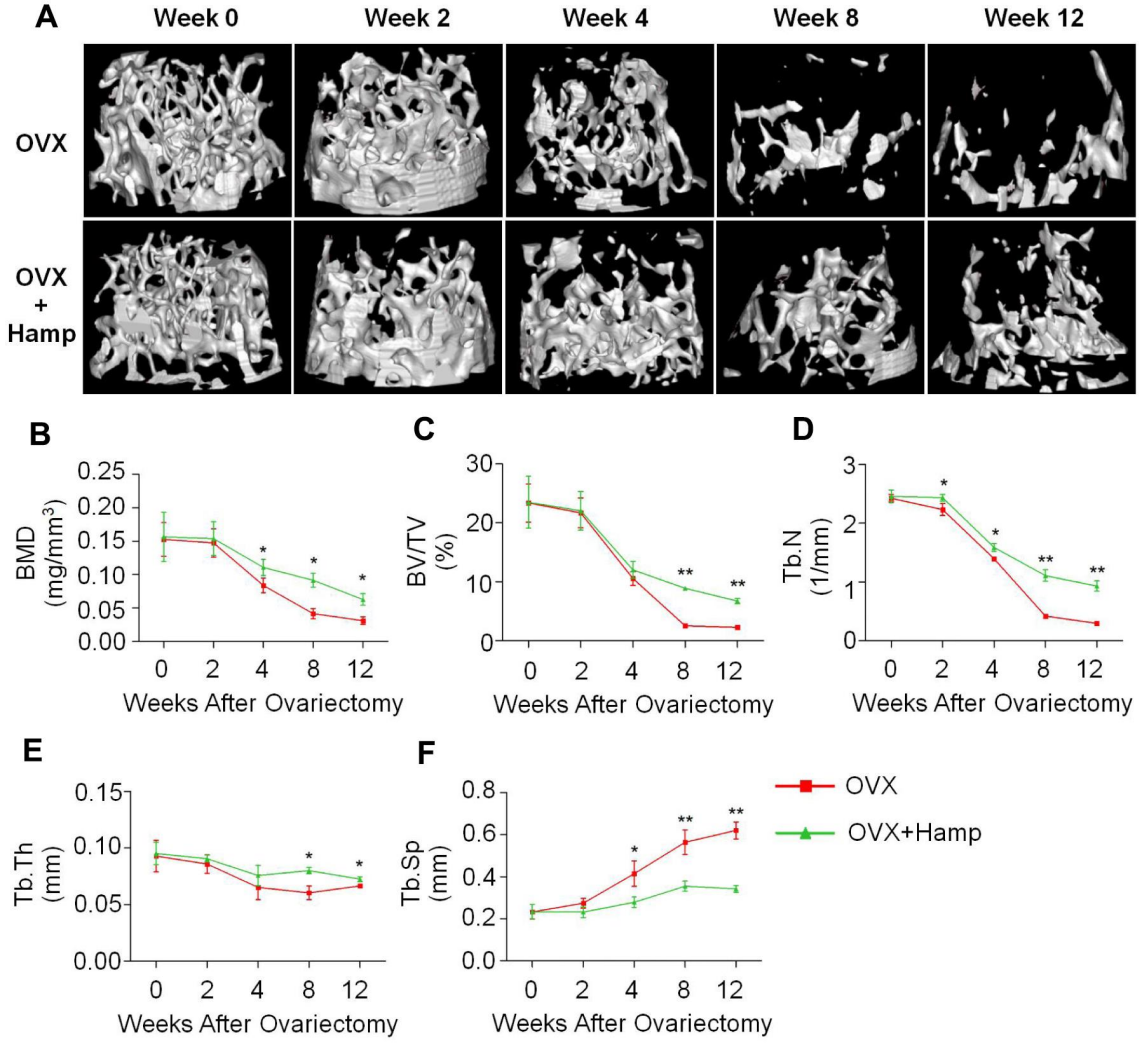
**Hepcidin overexpression (HAMP) of hepcidin rescues bone loss induced by ovariectomy in mice.** (**A**) The micro-CT showed that the loss of bone was rescued by overexpression of hepcidin in the OVX mouse. Micro-CT showed that (**B**) the distal femur bone mineral density and (**C**) the relevant parameters percent bone volume (BV/TV), (**D**) trabecular number (Tb.N), (**E**) trabecular thickness (Tb.Th) and (**F**) trabecular separation (Tb.Sp) were rescued by overexpression of hepcidin in the OVX mouse. Student's t-test was performed to determine statistic difference. The asterisks (*, **) indicate significant differences at P < 0.05, 0.01.

Micro-CT experiments were performed to observe mouse bone microstructure to assess bone mass rescued by hepcidin (Figure 3). The data showed that there was no significant change in femoral BMD in the two groups of mice at 4 weeks after ovariectomization (Figure 3A, 3B). From the 4th week, the bone loss of hepcidin overexpressing (HAMP) mice was greatly slower than that in non-overexpression group (Figure 3A, 3B). At week 12, the bone mass of mice in the overexpression hepcidin group was significantly higher than that of mice in the OVX group (Figure 3A, 3B). Meanwhile, trabecular bone related parameters like percent bone volume (BV/TV), trabecular thickness (Tb.Th), trabecular number (Tb.N) increased significantly whereas trabecular separation (Tb.Sp) was significantly reduced (Figure 3C–3F). To demonstrate whether hepcidin had other effect on bone metabolism, we compared the changes in non-OVX mice bone parameters between the hepcidin non-overexpression or overexpression. We did not observe significant difference in the two group mice bone (Supplementary Figure 2). These data showed that overexpression of hepcidin significantly rescued OVX mice bone loss by iron reduction.

### Over-expressed hepcidin had little effect on osteoblasts in OVX mice

Osteoblasts are the main cells involved in bone formation and bone remodeling. Excess iron was reported as an inhibitory element for osteoblasts [16, 26]. In order to study whether the effect of hepcidin on osteoporosis in ovariectomized mice is accomplished through osteoblasts, bone ALP staining experiments was performed to determine the number and status of osteoblasts in overexpressing mouse bone. It was found that, at the 8th week after ovariectomization, compared with mice without hepcidin overexpression, the number of osteoblasts showed a decreasing trend in hepcidin overexpression mice, but there was no significant statistical difference (Figure 2C, 2D). In order to further study whether hepcidin can affect the differentiation ability of osteoblasts in OVX mice, primary osteoblast were induced with mice serum after 14 or 21 days and then ALP and alizarin red staining were performed. The results showed that overexpression of hepcidin affected osteoblast differentiation activity (Figure 2E), but had no significant effect on the mineralization ability (Figure 2F). The expression of osteoblast-related genes was detected by qRT-PCR. After hepcidin overexpression, compared with the untreated group, the expression levels of *Alp* and *Bmp2* were significantly reduced in the overexpressed group (Figure 2G, 2H), but there was no statistical difference in *Runx2* and *Bglap* expression (Supplementary Figure 3). These data indicated that hepcidin had a lower effect on osteoblast number and differentiation in bilateral ovarian-removed mice.

### Overexpression of hepcidin decreased osteoclast number in OVX mice

**Figure 4:**
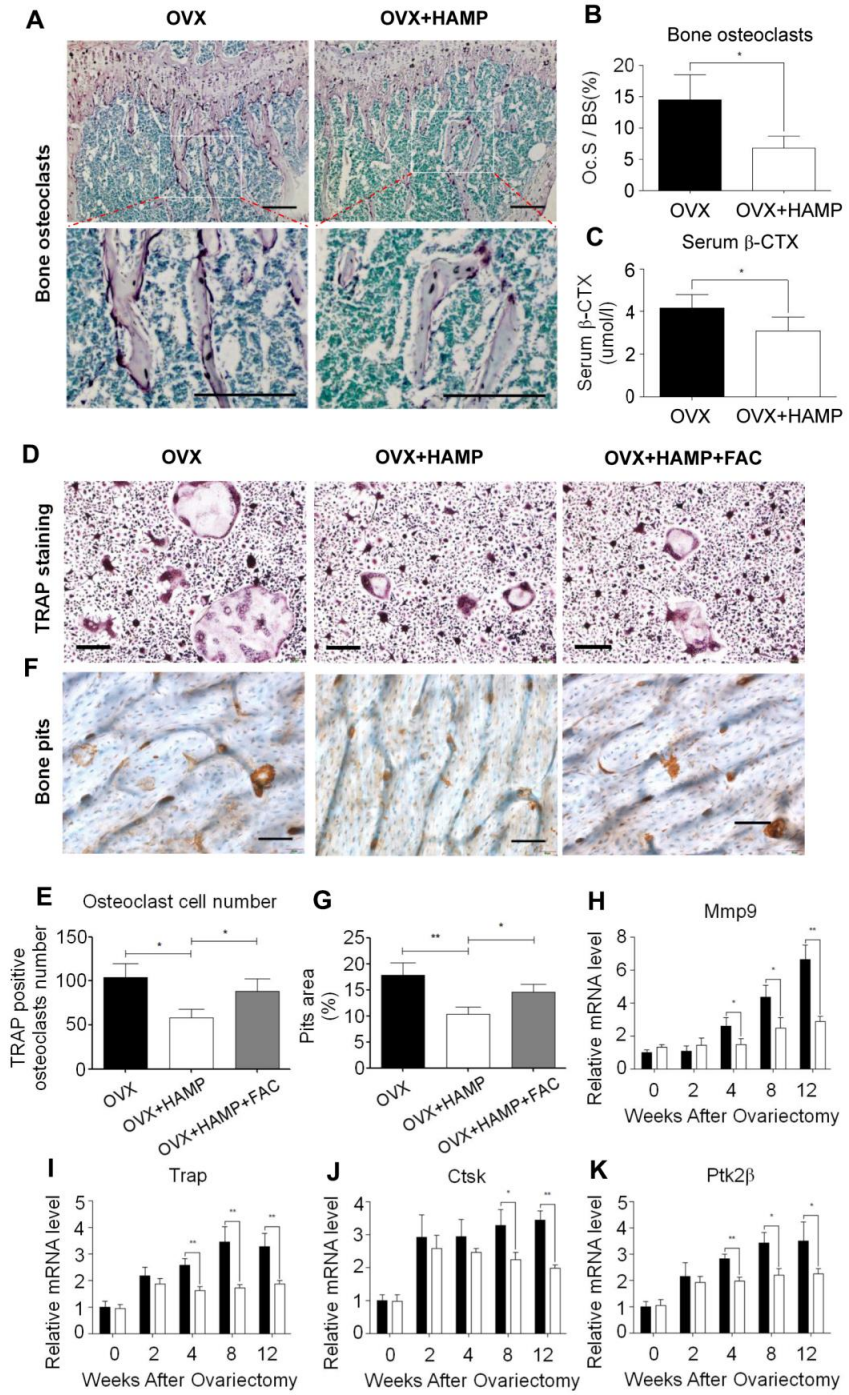
**Hepcidin overexpression inhibits osteoclast number and differentiate in the OVX mouse.** (**A**, **B**) TRAP staining shows that overexpression Hepcidin significantly inhibits the number of osteoclasts in femoral bone in the OVX mouse; (**C**) Overexpression hepcidin decrease CTX concentration in the OVX mouse serum. Bone marrow macrophage, which extracted from femur and cultured with M-CSF and RANKL for 8 days, stained with TRAP to assess its differentiation. (**D**) TRAP staining revealed that differentiation of osteoclasts *in vivo* M-SCF induced in the OVX mouse; (**E**) The osteoclasts were counted in the overexpression hepcidin and non overexpression hepcidin OVX mice; (**F**) The bone pits *in vivo* M-SCF induced osteoclasts, which was separate from OVX mouse; (**G**) The bone pits *in vivo* M-SCF induced osteoclasts, which was separate from overexpression hepcidin OVX mouse; Quantitative polymerase chain reaction (q-PCR) analysis of the expression of bone resorption markers, including (**H**) *Mmp9*, (**I**) *Trap*, (**J**) *Ctsk* and (**K**) *Ptk2β*. Scale bar, 200 μm. The asterisks (*, **) indicate significant differences at P < 0.05, 0.01.

Osteoclasts play an important role in bone remodeling. Previous studies found that estrogen, as an antioxidant hormone, can inhibit the proliferation and differentiation of osteoclasts. The abnormal activation of osteoclasts, compensatory proliferation of osteoblasts, and high bone turnover are the characteristics of bone loss in postmenopausal women [28]. To investigate the effect of hepcidin overexpression on osteoclasts, mice in the 8th week after ovariectomization were subjected to TRAP staining of bone sections to count the number of osteoclasts *in vivo*. It was found that osteoclasts were scattered on the surface of trabecular bone in the bone tissue of hepcidin over-expressed mice, whereas the osteoclasts were connected into pieces in the trabecular bone tissue in non-over-expressed mice (Figure 4A). In hepcidin over-expressed OVX mice, the level of osteoclasts were significantly reduced relative to mice that did not over-expression hepcidin (Figure 4B). β-CTX is a degradation product of β-collagen, and change in its serum concentration is an important indicator of osteoclast activity. ELISA was performed to detect the serum β-CTX concentration in experimental mice. The results showed the concentration of β-CTX in serum of hepcidin over-expressed OVX mice significantly decreased as compared to that in hepcidin non-over-expressed OVX mice (Figure 4C). These results indicated that overexpression of hepcidin reduced the number and activity of osteoclasts in OVX mice.

### Over-expressed hepcidin inhibited osteoclast differentiation in OVX mice

Osteoclasts are multinucleated cells formed by the fusion of monocytes, which perform the function of bone resorption. In order to verify whether overexpression of hepcidin affects the differentiation of osteoclasts, bone marrow macrophages of two groups of mice were isolated at different time points and cultured *in vitro*. Then they were induced into mature osteoclasts using M-CSF and RANKL and further subjected to TRAP staining and bone pits experiments. The results showed that TRAP-positive cells were significantly less in the over-expressed hepcidin group (Figure 4D, 4E). And the area of bone resorption pits of osteoclasts inoculated on the bone plate was significantly reduced in the hepcidin over-expressed group (Figure 4F, 4G). The number of osteoclasts and pits areas were partially recovered after FAC supplementation (Figure 4D–4G). In order to explore the reasons for the reduced differentiation ability of osteoclasts, qRT-PCR was used to detect the expression levels of osteoclast differentiation genes after induction at different time points. The expression levels of osteoclast differentiation marker genes (*Mmp-9*, *Trap*, *Ctsk*, *Ptk2β*) did not change after two weeks of hepcidin intervention, but after 4 weeks, the expression of osteoclast differentiation marker genes in the hepcidin over-expressed group was significantly down-regulated (Figure 4H–4K). These data indicated that the overexpression of hepcidin in OVX mice significantly reduced the differentiation ability of osteoclasts.

### Overexpression of hepcidin inhibited ROS production, mitochondrial biogenesis, and PGC-1β expression in osteoclasts

**Figure 5:**
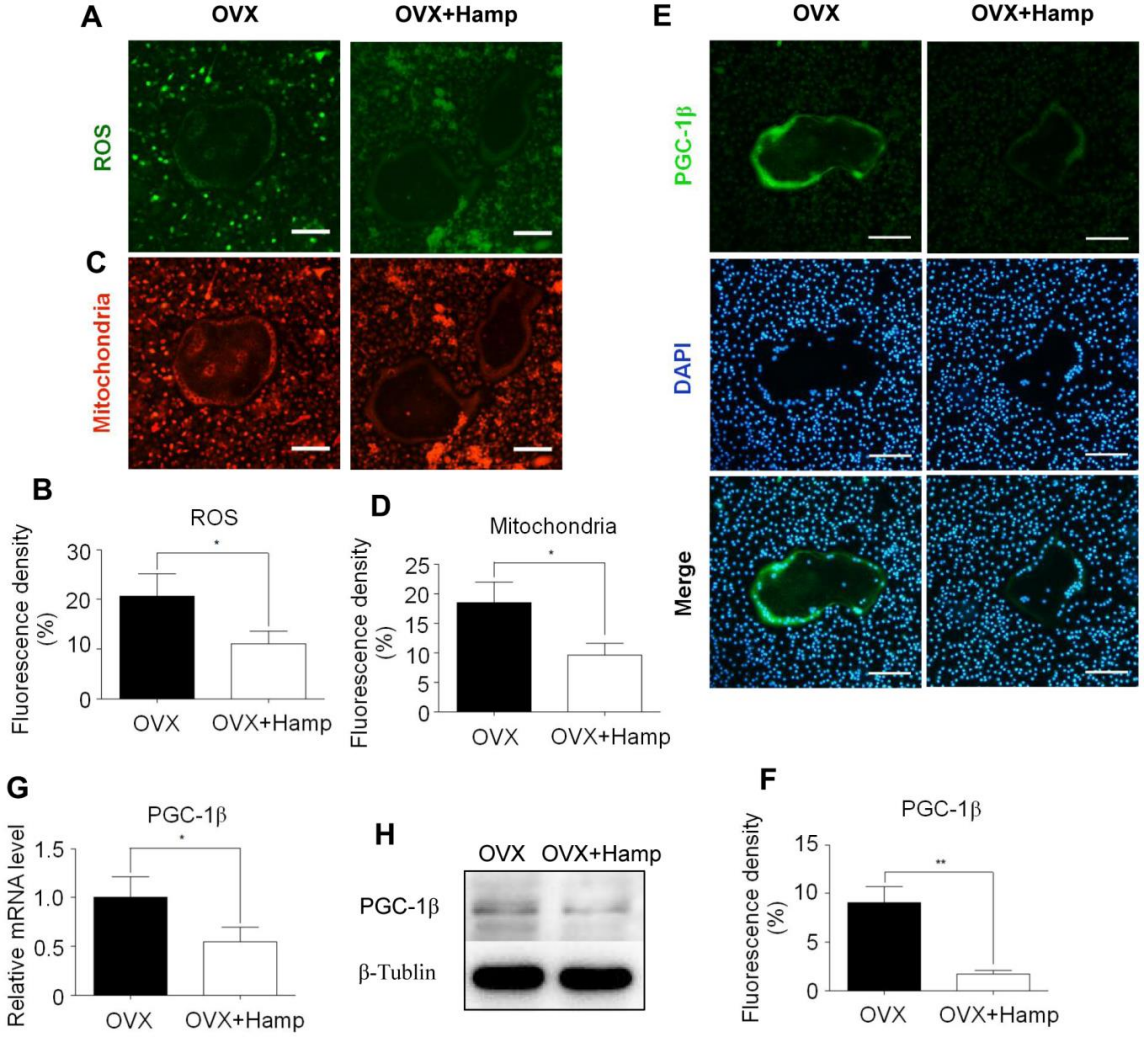
**Hepcidin overexpression inhibits ROS production, mitochondrial biogenesis, and PGC-1β expression in osteoclasts. Bone marrow macrophages were extracted from femur and cultured with M-CSF and RANKL for 8 days.** (**A**, **C**) The smaller cells are undifferentiated bone marrow macrophages and the larger cells in the middle are completely differentiated mature osteoclasts; (**A**) DCFH-DA and (**C**) mitochondrion-selective probes were used for assessing ROS and mitochondrial number in un-or-differentiated osteoclasts; (**B**, **D**) Mean fluorescence density of intermediate mature osteoclasts was measured to represent ROS and mitochondrial number respectively; (**E**) The smaller cells are undifferentiated bone marrow macrophages and the larger cells in the middle are completely differentiated mature osteoclasts; Immunocytofluorescence was used for assessing PGC-1β (**E**) in the osteoclasts, which were extracted from femur and cultured with M-CSF and RANKL for 8 days; (**F**) Mean fluorescence density of intermediate mature osteoclasts was measured to represent PGC-1β protein level; (**G**) the *PGC-1β* expression level was evaluated using qRT-PCR in osteoclasts; (**H**) PGC-1β protein levels were analyzed by western blotting in osteoclasts. Scale bar, 50 μm. The asterisks (*, **) indicate significant differences at P < 0.05, 0.01.

Previous studies have found that iron produces ROS and cooperates with PGC-1β to increase the number of mitochondria and activate osteoclast differentiation. Hepcidin overexpression inhibited osteoclasts by iron regulation. To verify whether hepcidin overexpression in OVX mice affects this process, mitochondrial staining and ROS detection experiments were performed in *in vitro*-induced osteoclasts. The results revealed that ROS production and the number of mitochondria were significantly reduced in cultured osteoclasts in hepcidin over-expressed group (Figure 5A–5D). Using immunocytofluorescence experiments, qRT-PCR, and western blotting it was found that the mRNA and protein levels of PGC-1β in the hepcidin over-expressed group were significantly reduced (Figure 5E–5H). These results indicated that overexpression of hepcidin reduced ROS production, mitochondrial synthesis, and PGC-1β expression in osteoclasts.

### Overexpression of hepcidin decreased PGC-1β expression in OVX mouse bone

**Figure 6:**
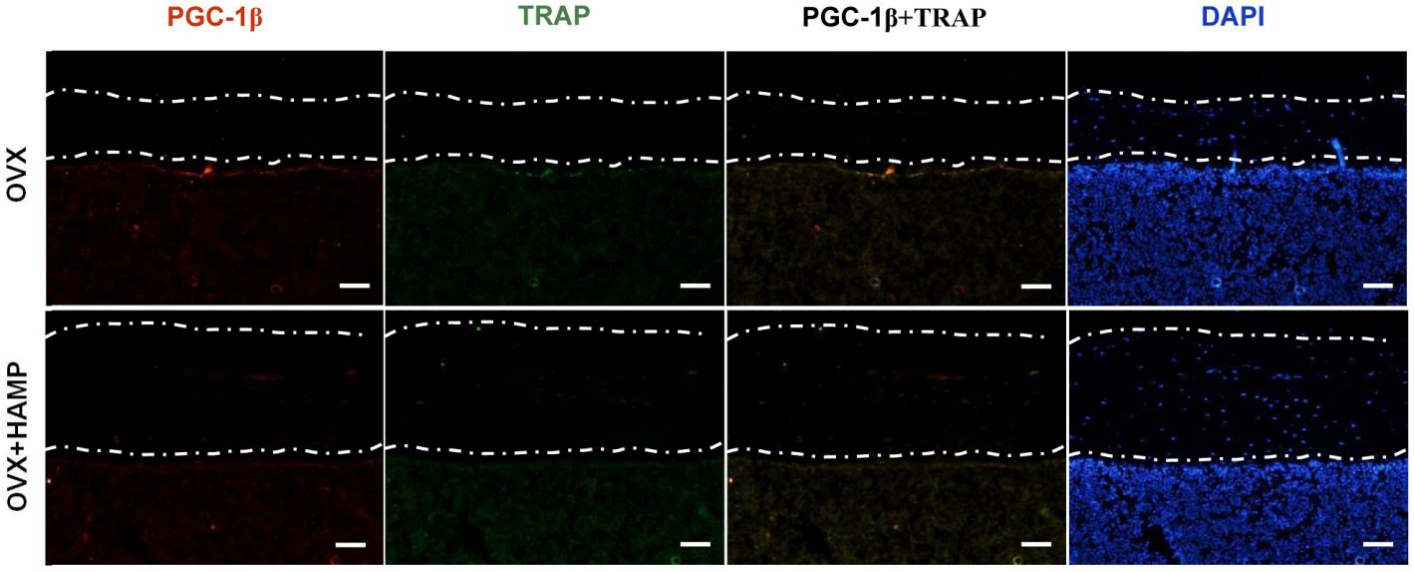
**Overexpression hepcidin decreases PGC-1β expression in OVX mouse bone.** The expression of PGC-1β in bone was evaluated by immunohistofluorescence assays in overexpression hepcidin and non-overexpression hepcidin OVX mouse bone. Scale bar, 50 μm.

In order to verify whether overexpression of hepcidin *in vivo* can reduce the expression of PGC-1β in the femur of OVX mice, immunohistofluorescence methods were used to detect the expression of PGC-1β in bone tissue. The abundance of PGC-1β and osteoclasts (TRAP^+^) was obviously increased under the bone cortex of the OVX+HAMP mouse femurs compared with that of the OVX mice (Figure 6). These results indicated that after OVX, hepcidin overexpression can reduce the expression of PGC-1β *in vivo*.

## DISCUSSION

Bone is a very active connective tissue. In the female population, estrogen is important for bone remodeling. Menopause destroys the balance of bone metabolism, which can lead to osteoporosis. In this study we found that in the postmenopausal female population with higher hepcidin, bone masses were also elevated and there was a positive correlation between the hepcidin level and bone mineral density. Through the negative regulation of iron, hepcidin restricted the use of iron by osteoclasts, down-regulated ROS and PGC-1β, inhibited mitochondrial biogenesis, its differentiation and bone resorption function, and thereby significantly improved bone mass. The tendency of bone loss was observed to be suppressed in ovariectomized mice overexpressing the hepcidin gene.

Osteoporosis results from multiple causes, and the current treatment strategies cannot provide satisfactory treatment to all patients with osteoporosis. Therefore, the research on osteoporosis is becoming increasingly abundant. One of the methods for the treatment of postmenopausal osteoporosis, hormone replacement therapy, has a miraculous effect on the disease, but a large number of side effects that limits the implement of this treatment [29–31]. Although some studies suggested that dose control did not increase the incidence of breast and cervical cancer, osteoporosis patients with breast cancer have been prohibited from using hormone replacement therapy [32]. Other studies have found that the cause of postmenopausal osteoporosis was the abnormal activation of osteoclasts, and as the osteogenic drug PTH cannot alleviate osteolysis, it was not suitable for the treatment of postmenopausal osteoporosis [33]. Therefore, it could be of great clinical significance to find a supplementary treatment method for postmenopausal osteoporosis.

Iron accumulation is a risk factor for accelerated bone loss in postmenopausal women. Hepcidin is an important regulator of iron homeostasis in the body. Clinical data revealed that with the decrease of hepcidin, BMD showed a significant downward trend. It has been speculated that overexpression of hepcidin may have a protective effect on postmenopausal osteoporosis. Previous studies have found chronic iron accumulation and significant bone loss in mice 7 months after hepcidin knockout [26]. In contrast, supplementation with hepcidin and reduction in ferritin protected against femur bone loss in these mice [34]. In this study, the levels of ferritin and bone iron in mice were reduced after hepcidin overexpression, which was consistent with the results of previous studies [35, 36]. Overexpression of hepcidin significantly protected bone mass and BMD caused by OVX. This demonstrated that hepcidin overexpression can protect against osteoporosis caused by estrogen deficiency.

In bone metabolism, the balance of osteoblasts and osteoclasts is very important to maintain the homeostasis. Regulation of bone mass by hepcidin may involve regulating the function of these two cells to protect the bones of OVX mice. A previous research by our group found that hepcidin-FPN1 was present in osteoblasts [37]. After overexpressing hepcidin, it was observed that there was no statistically significant decrease in the number of osteoblasts in OVX mouse bone tissue, and there was no significant difference in the differentiation of osteoblasts cultured *in vitro*. These results indicated that the protective effect of hepcidin on postmenopausal osteoporosis was not achieved by increasing the process of osteogenesis.

Osteoclasts play an important role in bone remodeling and bone metabolism. Postmenopausal osteoporosis is also mainly caused by increased osteoclast activity. In this study, osteoclast differentiation and proliferation were significantly reduced after hepcidin overexpression, indicating that hepcidin protected postmenopausal osteoporosis by affecting osteoclasts. Our team and other groups proved that iron can enhance the differentiation and proliferation of osteoclasts by generating ROS, and NF-κB signaling pathway plays an important role in this process [17, 38]. As early as 2009, Kiyo-aki et al. found that PGC-1β and iron absorption cooperatively promote mitochondrial biogenesis to activate osteoclast differentiation [19]. In order to verify this information, we performed *in vitro* experiments to detect the correlation between expression of PGC-1β and mitochondrial number. It was found that the expression of PGC-1β was significantly down-regulated in osteoclasts in both *in vitro* and *in vivo* experiments after hepcidin overexpression in OVX mouse. Excessive mitochondrial biogenesis leads to more ROS production, which in turn enhances the expression of PGC-1β and osteoclast marker gene NFATc1 through the phosphorylation of CREB, further enhancing osteoclast differentiation [19]. Overexpression of hepcidin reduces the iron concentration in bone tissue. Decreasing the supply of iron might be the reason for reducing osteoclast differentiation and bone resorption. The results obtained for osteoclast cells isolated from hepcidin overexpressing mice also confirmed.

Hepcidin reduces iron levels and improves postmenopausal osteoporosis, as evidenced in this study. Therefore, additional alternative mechanisms that explain hepcidin-mediated regulation of iron levels warrant further investigation. Studies have reported that the estrogen receptors also regulate the expression of cytochromes P450 (CYP), the heme-containing enzymes. CYP metabolism may considerably affect iron availability by altering iron or hormone levels associated with menopause [39, 40]. Additionally, the vitamin D hormone is also known to regulate the expression of antimicrobial peptides, including cathelicidin and hepcidin, by the retinoid X receptor (RXR) [41]. Therefore, vitamin D hormone status may also play a role in regulating iron and or bone mineralization phenotypes in post-menopausal women.

This study has several limitations. First, although the population cohort data showed that hepcidin could ameliorate post-menopausal osteoporosis, the number of cases was limited and this was a single-center study. Second, although we detected the BMD by micro-CT, because no mechanical test data were available in this study, it is hard to accurately assess whether the increased bone density could improve bone quality.

**Figure 7:**
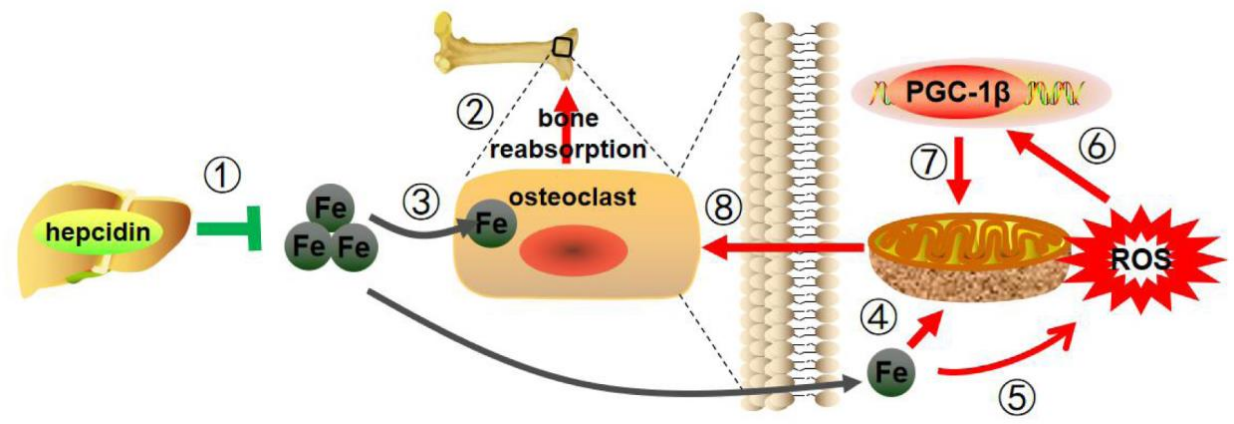
**Mechanism pattern diagram of hepcidin overexpression to improve bone metabolism.** Hepcidin overexpression in the model mice liver (1) reduces estrogen deficiency-induced bone loss (2), through reducing iron content (3), decreasing mitochondrial respiration (4) and ROS production (5), suppressing PGC-1β expression (6), inhibiting mitochondrial biogenesis (7), and depressing the function of osteoclasts (8).

In this study, hepcidin overexpression was used to study osteoporosis caused by estrogen deficiency in a mouse model. As illustrated in Figure 7, hepcidin could regulate iron metabolism levels and regulate osteoclasts through PGC-1β, thus had a protective effect on postmenopausal osteoporosis. This study provides a new theoretical basis for hepcidin to regulate bone metabolism and also provides a new candidate therapeutic target for the clinical treatment of postmenopausal osteoporosis.

## MATERIALS AND METHODS

### Study participants

The study participants consisted of subjects aged 55 years or older (55-74 years) who had undergone health examinations at Second Affiliated Hospital of Soochow University, and their BMD was measured. Because of the lack of hepcidin test data, we collected the serum of the participants and detected the level of hepcidin with Elisa kit (DRG Instrument GmbH, EIA-5782) and this process was approved by the institutional review board of the Second Affiliated Hospital of Soochow University (ethic approval number: JD-LK-2020-028-01). The participants were mostly healthy without malignancy, diabetes, hepatitis, Alzheimer’s disease and other age-related diseases.

### Animals

Hepcidin conditional overexpression mice used in the experiment were obtained from “Cambridge-Soochow University Genome Resource Center (CAM-SU GRC). These mice were bred and maintained in specific pathogen free (SPF) laboratory in Soochow University, where conditions were maintained at 25° C, 45-55% relative humidity and 12h-light, 12h-dark with free access to food and the weakly acidic tap water. Bilateral ovaries were removed at the age of 8 weeks in all mice. These mice were injected intraperitoneally with 10 mg/mL tamoxifen for another 5 days for inducing hepcidin overexpression in the liver [42]. The hepcidin gene of hepcidin conditional overexpression mice (group OVX + HAMP) was sensitive to tamoxifen and easy to induce, whereas the OVX group mice were insensitive. Twelve weeks later, mice were sacrificed and related specimens were collected in 0 week (the day of ovariectomy), 2 weeks, 4 weeks, 8 weeks, 12 weeks, respectively. All animal experiments were approved by the animal care committee of Soochow University and conformed to all relevant regulatory standards (ethic approval number: ECSU-201800093).

### Mice serum assay

A blood volume of 0.7-1.0 mL was obtained by cardiac puncture and poured into 1.5 mL EP tube. Then all mice blood was put in a centrifuge at 3000 rpm, 4° C for 20min and centrifuged twice. Each time supernatant was collected. Finally, the collected serum specimens were placed in -80° C refrigerator until use. In order to analyze serum estrogen (E2, YH, YH9009), ferritin (Abcam, ab157713), hepcidin (ElabScoemce, E-EL-M0671c) and β-CTX (CUSABIO, CSB-E12782m) levels, frozen blood serum samples were melted on ice and measured by enzyme-linked immunosorbent assay (ELISA) kit.

### Perls staining of liver and bone

To observe ferric iron deposits, Perls staining was carried out on liver tissues and decalcified tibia. The complete method has been described elsewhere. Briefly, the right tibia bones were embedded in resin and sections (6 μm thick) were cut by the department of orthopedics Research Institute of Soochow University. Liver tissues were fixed in 4% PFA buffered and embedded in paraffin and cut in 5 μm thick sections. Perls staining was carried out on decalcified tibias and liver tissues and visualized by light microscopy.

### Micro-computed tomography (micro-CT) assay

Right femora bones of mice were taken and the soft tissue around the bone was removed. These bones were fixed in 4% polyformaldehyde (PFA) solution and kept in 4° C for 48 hours. To estimate the change of bone microarchitecture, 1176 micro-computed tomography scanner (Skyscan, Belgium) with 9-mm voxel size, 59 KVp, 127 μA, and 0.48 rotation step, was used to measure the distal region of the femur from 60 mm approximately to the end of the distal growth plate over 1.7 mm toward the diaphysis. The following trabecular parameters were analyzed: bone mineral density (BMD), percent bone volume (BV/TV), trabecular thickness (Tb.Th), trabecular number (Tb.N) and trabecular separation (Tb.Sp).

### Primary osteoblast cell cultures and alkaline phosphatase (ALP) and alizarin red staining

Primary osteoblasts harvested from one or two-day-old newborn mice calvaria, were washed with 75% ethanol for 5 min and PBS repeatedly to remove residual blood and adipose tissue. The calvaria were cut into 2-5 mm^2^ fragments and digested with 0.25% trypsin for 15 min to remove the fibrous tissue cells. The digestions were discarded, and then 0.1% of type II collagenase was added to 10 ml at 37° C for 20 minutes to digest. The samples were centrifuged at 3000 rpm for 3 minutes at room temperature. Discarding the supernatant, osteoblast cells were collected and incubated for 7 days with alpha-MEM. 10% fetal bovine serum (FBS) in alpha-MEM was changed with mice serum which was obtained from 8 weeks OVX or OVX + HAMP mice before and osteoblasts were cultured for further 14 or 21 days. To visualize osteoblasts ALP and mineralization activity, these cells were fixed in 3.7% formaldehyde for 10 min, and analyzed using alkaline phosphatase and alizarin red staining with ALP kit (Solarbio, G1480) and alizarin red kit (Solarbio, G3280) following the manufacturer’s instructions.

### Primary osteoclast cell cultures and tartrate-resistant acid phosphatase (TRAP) staining

The left femora were moved out and the soft tissues were discarded. Each end of bone was opened after being soaked in 75% alcohol for 10min. Finally, the bone marrow was pulled out and filtered using 200 mesh filters. These cells were placed at 37° C in 5% CO_2_ standard environment incubator for 12-16 h. The next day, M-CSF (PeproTech, 315-02) was added to the supernatant and the concentration was adjusted to 50 ng/mL. Three days later, the supernatant was discarded, while M-CSF and RANKL (PeproTech, 315-11C) were added. 200 μM ferric ammonium citrate (FAC) was supplied to cells during the first days of culture [15]. Depending on the growth of cells, fluid was changed every one or two days. Osteoclast cells were formed within another 4-6 days. To visualize the osteoclast’s TRAP activity, they were fixed in 3.7% formaldehyde for 10 min, then analyzed using tartrate-resistant acid phosphatase (TRAP) staining with Leukocyte Acid Phosphatase Assay kit (Sigma, 387A) following the manufacturer’s instructions. TRAP-positive osteoclast cells were visualized by light microscopy.

### Pit formation assay

To compare the osteoclast reabsorption ability of mice in different treatment groups, the pit formation assay was performed. Sterilized bovine cortical bone slices were placed in 24-well plate and primary osteoclast cell cultures as before. After an incubation of 8 days, the bone slices were brushed with a soft brush to remove any cell remains and again incubated with 20 μg/mL peroxidase-conjugated WGA (Wheat germ agglutinin) lectin for 30-60 min at room temperature. After washing in PBS twice, bone chips were incubated with 0.52 mg/mL 3,3’-diaminobenzidine (DAB) and 0.03% H_2_O_2_ for 30 minutes. Samples were mounted with 50% glycerol/PBS and photo graphed by an Olympus digital camera. The areas of pits formed on the slices were counted by Image-Pro Plus 6.0 (IPP) software (UVP, America).

### Histochemical staining for TRAP and ALP

In order to visualize mature osteoblasts and osteoclasts activity in bones, the right femora specimens used for micro-computed tomography scanning were decalcified in 0.5 M EDTA solution for 4 weeks with the change of solution twice a week. Then decalcified bone specimens were dehydrated in 70% ethanol for 1h, 80% ethanol for 2h, 90% ethanol for 1h, and 100% ethanol for 2h followed by vitrification by dimethylbenzene before embedding in paraffin. Finally, sections (4μm thick) were cut and mounted on adhesive slides. The method of ALP and TRAP staining were similar as performed before. The osteoblast surface/bone surface (Ob.S/BS) was the ratio of the ALP-stained area to total bone area and the osteoclast surface/bone surface (Oc.S/BS) was the ratio of the TRAP-stained area to the total bone area [43], which were quantified using Imagej software.

### Quantitative real-time PCR (qRT-PCR)

Total RNA was extracted from mature primary osteoblasts or osteoclasts with TRIzol and reverse transcribed into cDNA using a reverse transcription kit (Invitrogen, AM1710). qRT-PCR was performed with real time PCR System (Life Technologies, ViiA7) at following temperatures: 95° C for 5 seconds, 95° C for 5 seconds and 60° C for 31 seconds for a total of 40 cycles. All primers were purchased from GENEWIZ. The primers used are listed in Supplementary Table 2. The levels of gene expression were normalized by the expression level of β-actin.

### Measurement of intracellular ROS and mitochondria level

Primary osteoclast cells were cultured and treated in 24-well plates. On the last day of culture, cells were incubated in the dark at 37° C in serum-free medium containing 10 mM DCFH-DA probes (Beyotime, S0033) and 100 nM MitoTracker® Deep Red FM probes (Invitrogen, M22426) for 20 min. The cells were washed three times in serum-free medium to remove extracellular probes. Cells were immediately examined using a fluorescent microscope.

### Immunocytofluorescence and immunohistofluorescence analysis

The mature osteoclasts were fixed with 4% PFA, permeabilized with 0.1% triton X-100, and blocked with 5% bovine serum albumin. Cells were incubated overnight at 4° C with mouse anti-Pgc-1β (1:1000, Santa Cruz, sc-373771) primary antibody, followed by staining with Alexa Fluor 488 anti-mouse secondary antibody. Cells were immediately examined using a fluorescent microscope.

The bone sections were deparaffinized in xylene and rehydrated. Antigen retrieval was performed with protease K at 37° C for 30 min. A solution of 3% H_2_O_2_ was used to block the activity of endogenous peroxidase. The sections were then incubated overnight at 4° C with primary antibody mouse anti-Pgc-1β (1:200, Santa Cruz, sc-373771) (1:200) and rabbit anti-TRAP (1:300, Abcam, ab133238), followed by staining with Alexa Fluor 488 anti-mouse and Alexa Fluor cy3 secondary antibody. The bone sections analyzed with a microscope.

### Western blot analysis

To analyze the level of PGC-1β, mature primary osteoclasts proteins were extracted. A total of 40 mg of protein was mixed with 5×SDS–PAGE sample loading buffer and boiled at 95° C for 3 min. Proteins were transferred to PVDF membranes by electroblotting. The membranes were blocked using 5% non-fat dry milk in TBST for 0.5 h and then probed with anti-Pgc-1β (1:500) and anti-β-tubulin (1:3000, Abcam, ab6160). Primary antibodies were detected using HRP labeled antibody to Rabbit/Mouse IgG (HtL) (1:10000). Bound complexes were measured using the Odyssey Infrared Imaging System.

### Statistical analysis

Data were expressed as mean ± SD from at least three independent experiments. Statistical analyses were performed with SPSS 22.2 software and used two-tailed Student’s t-test to analyze differences between groups. P < 0.05 was considered statistically significance.

## Supplementary Material

Supplementary Figures

Supplementary Tables
