# Supplementary material for: Hepcidin-induced reduction in iron content and PGC-1β expression negatively regulates osteoclast differentiation to play a protective role in postmenopausal osteoporosis
Source: Aging (Albany NY). 2021 Apr 4;13(8):11296–314. doi: 10.18632/aging.202817 (PMC8109081; doi:10.18632/aging.202817)
Supplement: Supplementary Figures [file aging-13-202817-s001.pdf]

SUPPLEMENTARY FIGURES

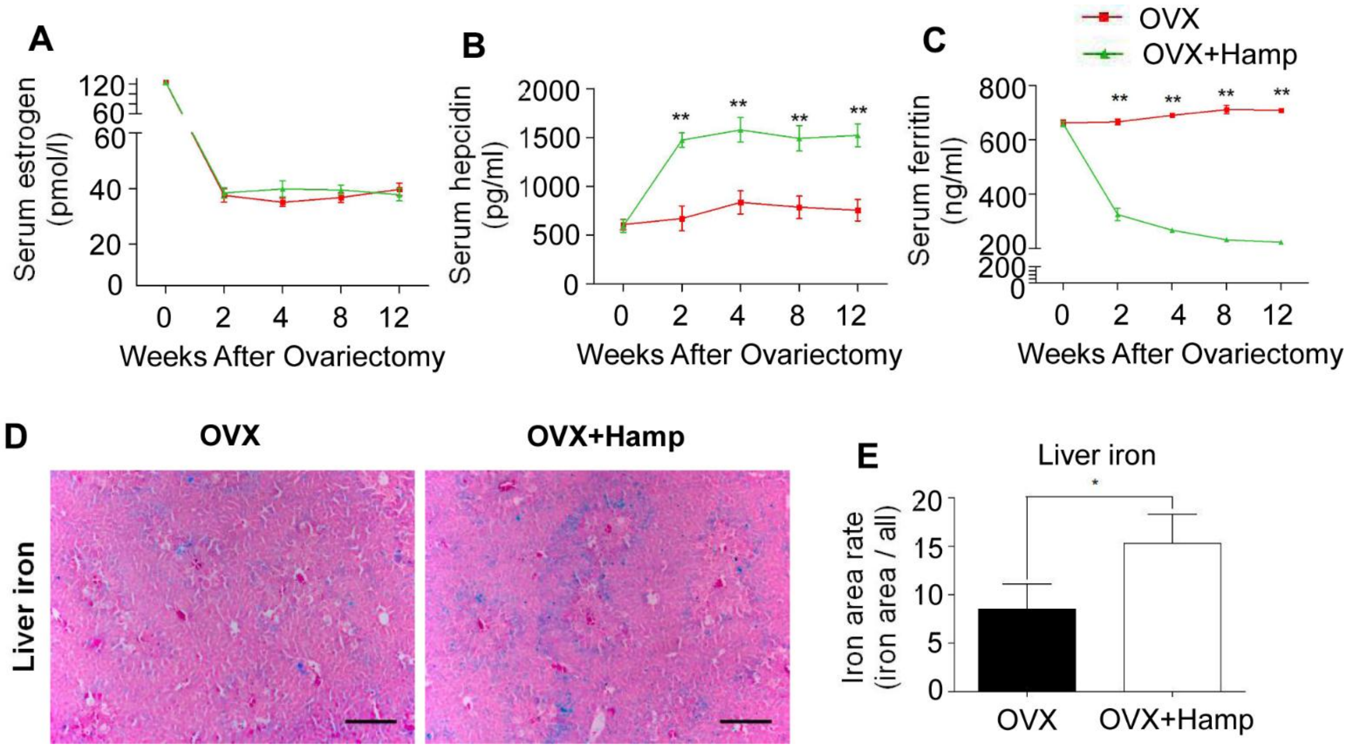

Supplementary Figure 1. Hepcidin overexpression modulates the iron metabolism. (**A**) Hepcidin overexpression has no effect on serum estrogen level; (**B**) Hepcidin overexpression increased the serum hepcidin level; (**C**) Hepcidin over-expression increased the serum ferrtin level. Liver slices stained with prussian blue to evaluate iron deposit; (**D**) Hepcidin overexpression increase the irons contents in the mouse liver. (**E**) Quantitation of the mouse liver iron deposit from (**D**). Scale bar, 200 µm. The asterisks (*, **) indicate significant differences at P < 0.05, 0.01.

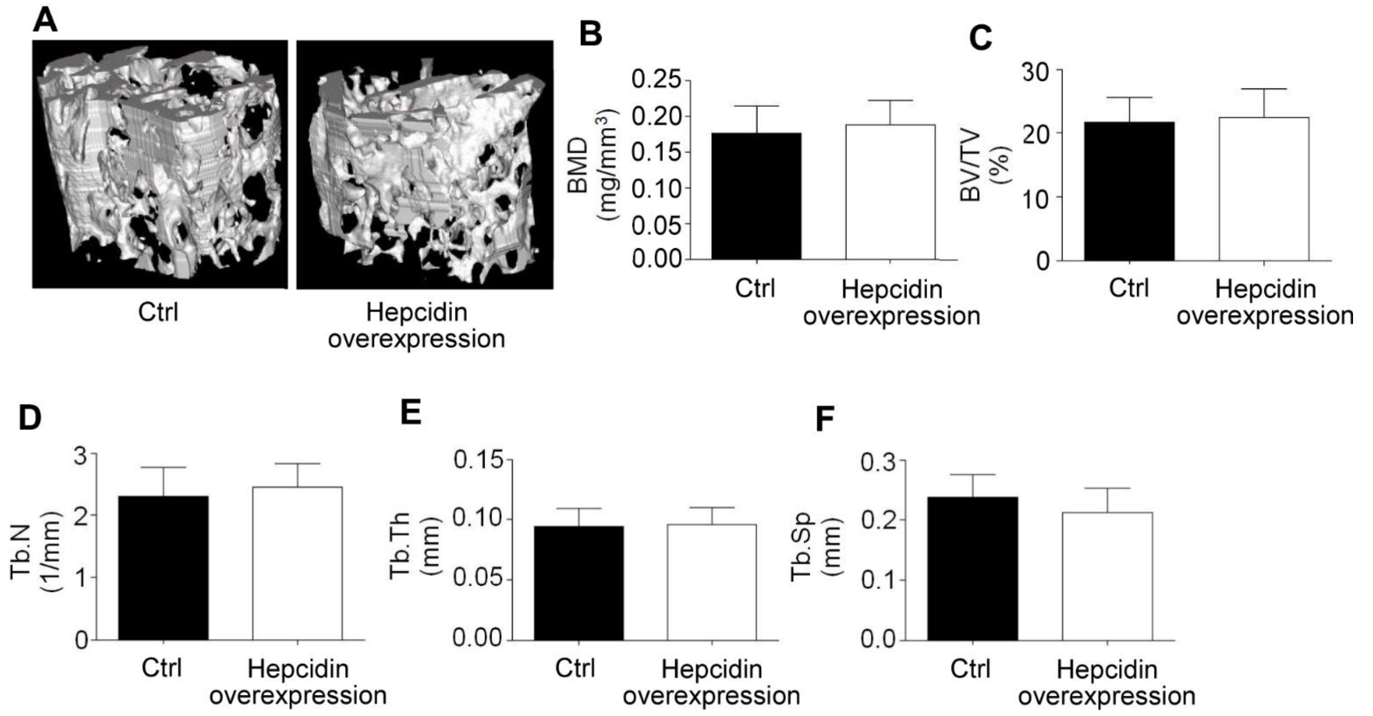

Supplementary Figure 2. Hepcidin overexpression has no effect on non-ovariectomied mice bone. (**A**) The micro-CT showed that there is no significant difference between the bone of the tamoxifen treated non-OVX mice; Micro-CT showed that (**B**) the distal femur bone mineral density, (**C**) the relevant parameters percent bone volume (BV/TV), (**D**) trabecular number (Tb.N), (**E**) trabecular thickness (Tb.Th) and (**F**) trabecular separation (Tb.Sp) are no difference between the bone parameters of the hepcidin overexpression non-OVX mice.

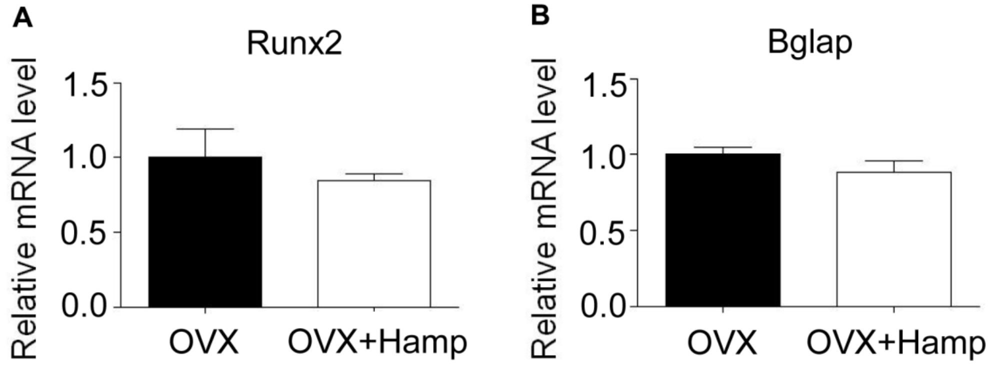

Supplementary Figure 3. Hepcdin overexpression has no effect on osteoblast marker gene. (**A**, **B**) Quantitative polymerase chain reaction (q-PCR) analysis of the expression of bone formation markers including (**A**) Runx2, (**B**) Bglap.
