# Supplementary material for: Hepcidin-induced reduction in iron content and PGC-1β expression negatively regulates osteoclast differentiation to play a protective role in postmenopausal osteoporosis
Source: Aging (Albany NY). 2021 Apr 4;13(8):11296–314. doi: 10.18632/aging.202817 (PMC8109081; doi:10.18632/aging.202817)
Supplement: Supplementary Tables [file aging-13-202817-s002.pdf]

SUPPLEMENTARY TABLES

Supplementary Table 1. Baseline characteristics of the study population.

Variables	Total (n=35)	Bone mineral density normal (or reduce) women (n=27)	Osteoporosis women (n=8)
**Hepcidin(pg/ml)**	26.01±10.35	28.43±9.84	17.34±7.51
**lumbar BMD (g/cm2)**	0.92±0.12	0.97±0.09	0.76±0.04
**collum femoris BMD (g/cm2)**	0.79±0.11	0.83±0.11	0.71±0.06
**Age (years)**	62.31±5.79	62.11±6.01	62.11±5.64
**Fasting glucose (mmol/L)**	5.18±0.56	5.18±0.55	5.10±0.63
**HbA1c (%)**	5.69±0.34	5.76±0.36	5.49±0.22
**HDL cholesterol (mmol/L)**	1.42±0.34	1.43±0.36	1.38±0.24
**LDL cholesterol (mmol/L)**	3.30±0.75	3.33±0.78	3.18±0.63
**ALT (U/L)**	19.18±10.57	18.58±7.97	20.33±16.22
**AST (U/L)**	21.29±6.32	20.77±6.00	22.89±6.99
**Urea**	5.34±1.32	5.41±1.42	5.14±0.93
**Creatinine**	60.24±9.34	61.27±9.37	58.22±9.31
**Lithic acid (µmol/L)**	298.85±90.10	308.58±94.16	266.67±66.92
**Ferritin (ng/mL)**	175.96±91.16	151.99±78.92	256.87±82.76

BMD = bone mineral density; HbA1c = glycated hemoglobin; HDL = high-density lipoprotein; LDL = low-density lipoprotein; ALT = alanine aminotransferase; AST = aspartate aminotransferase.

Supplementary Table 2. Primers used for quantitative RT-PCR.

Gene	Primers (Forward/Reverse)
**Bmp2**	**5'-AAGCGTCAAGCCAAACACAAAC-3'**
	**5'-GCCACGATCCAGTCATTCCAC-3'**
**Alp**	**5'-CCAACTCTTTTGTGCCAGAGA-3'**
	**5'-GGCTACATTGGTGTTGAGCTTTT-3'**
**Runx2**	**5'-AACTTCCTGTGCTCCGTGCTG-3'**
	**5'-TCGTTGAACCTGGCTACTTGG-3'**
**Bglap**	**5'-GGACCATCTTTCTGCTCACTCTG-3'**
	**5'-GTTCACTACCTTATTGCCCTCCTG-3'**
**Mmp9**	**5'-TCCAGTACCAAGACAAAG-3'**
	**5'-TTGCACTGCACGGTTGAA-3'**
**Trap**	**5'-TACCTGTGTGGACATGACC-3'**
	**5'-CAGATCCATAGTGAAACCGC-3'**
**Ptk2β**	**5'-CGTCTTCCTTCTCCACATCC-3'**
	**5'-TTGAGCCGTGTAAAAGTGGG-3'**
**CTSK**	**5'-GCCGTGGCGTTATACATACA-3'**
	**5'-CTTCCAATACGTGCAGCAGA-3'**
**Pgc-1β**	**5'-AAGAACTTCAGACGTGAGAGCAGAG-3'**
	**5'-GCATGCCGGACGCTTG-3'**
**β-actin**	**5'-TCCTGTGGCATCCACGAAACT-3'**
	**5'-GAAGCATTTGCGGTGGACGAT-3'**
